# Impact of mutational studies on the diagnosis and the outcome of high-risk myelodysplastic syndromes and secondary acute myeloid leukemia patients treated with 5-azacytidine

**DOI:** 10.18632/oncotarget.25046

**Published:** 2018-04-10

**Authors:** Marta Cabezón, Joan Bargay, Blanca Xicoy, Olga García, Josep Borrás, Mar Tormo, Sílvia Marcé, Carme Pedro, David Valcárcel, Maria-José Jiménez, Ramón Guàrdia, Laura Palomo, Salut Brunet, Ferran Vall-Llovera, Antoni Garcia, Evarist Feliu, Lurdes Zamora, On Behalf of the CETLAM Group

**Affiliations:** ^1^ Hematology Service, ICO Badalona-Hospital Germans Trias i Pujol, Josep Carreras Leukaemia Research Institute, Universitat Autònoma de Barcelona, Badalona, Spain; ^2^ Departament de Medicina, Universitat Autònoma de Barcelona, Badalona, Spain; ^3^ Hematology Service, Hospital Son Llàtzer, Mallorca, Spain; ^4^ Josep Carreras Leukemia Research Institute, Campus Germans Trias i Pujol, Universitat Autònoma de Barcelona, Badalona, Spain; ^5^ Hematology Service, Hospital Clínic de Valencia, Valencia, Spain; ^6^ Hematology Service, Hospital del Mar, Barcelona, Spain; ^7^ Hematology Service, Hospital Vall d’Hebron, Barcelona, Spain; ^8^ Hematology Service, ICO Girona-Hospital Josep Trueta, Girona, Spain; ^9^ Hematology Service, Hospital de Sant Pau, Barcelona, Spain; ^10^ Hematology Service, Hospital Mútua de Terrassa, Terrassa, Spain; ^11^ Hematology Service, Hospital Arnau de Vilanova, Lleida, Spain

**Keywords:** myelodysplastic syndromes, secondary acute myeloid leukemia, targeted deep sequencing, prognostic factors, 5-azacytidine

## Abstract

Myelodysplastic syndromes (MDS) are stem cell disorders caused by various gene abnormalities. We performed targeted deep sequencing in 39 patients with high-risk MDS and secondary acute myeloid leukemia (sAML) at diagnosis and follow-up (response and/or relapse), with the aim to define their mutational status, to establish if specific mutations are biomarkers of response to 5-azacytidine (AZA) and/or may have impact on survival. Overall, 95% of patients harbored at least one mutation. *TP53, DNMT3A* and *SRSF2* were the most frequently altered genes. Mutations in *TP53* correlated with higher risk features and shorter overall survival (OS) and progression free survival (PFS) in univariate analysis. Patients with *SRSF2* mutations were associated with better OS and PFS. Response rate was 55%; but we could not correlate the presence of *TET2* and *TP53* mutations with AZA response. Patients with sAML presented more variations than patients with high-risk MDS, and usually at relapse the number of mutations increased, supporting the idea that in advanced stages of the disease there is a greater genomic complexity. These results confirm that mutation analysis can add prognostic value to high-risk MDS and sAML patients, not only at diagnosis but also at follow-up.

## INTRODUCTION

Myelodysplastic syndromes (MDS) are a group of myeloid neoplasms originated in hematopoietic stem cells, characterized by cytopenias, dysplasia in one or more cell lines, ineffective hematopoiesis and an increased risk of progression to secondary acute myeloid leukemia (sAML) [1, 2]. The outcome of MDS patients is extremely variable with median overall survival (OS) ranging from over 5 years to less than 6 months [3].

Recurrent chromosomal aberrations have been linked with distinct outcomes and are one of the most important risk factors when patients are stratified according to their risk level before therapy. However, approximately 50% of patients with AML or MDS have a normal karyotype and lack recurrent cytogenetic abnormalities, which suggests the implication of other molecular events in the pathogenesis of these diseases [4].

Over the past decade, the application of new high-throughput technologies to the study of MDS has led to the identification of several recurrently mutated genes in these disorders [5–7] and somatic gene mutations have been found to be more common than previously expected. The most common mutations found in MDS occur in genes involved in RNA splicing (including *SF3B1*, *SRSF2*, *U2AF1* and *ZRSR2*) [8] and epigenetic regulators (including *TET2*, *ASXL1* and *DNMT3A*) [9]. Several regulators of signal transduction (*NRAS*, *JAK2*) and transcription factors (*RUNX1*, *TP53*) are also frequently mutated in MDS. The complex patterns of associations between gene mutations have revealed epistatic interactions between spliceosome components and epigenetic modifiers in MDS [10].

Some of the mutated genes identified using these high-throughput techniques have been shown to provide important prognostic information. For example, mutations in *ASXL1*, *TP53*, *EZH2*, *ETV6* and *RUNX1* have been described to be predictors of poor OS in patients with MDS, independently of the already established risk factors [11].

This genetic variability together with the diversity in the clinical presentation of MDS, emphasizes the need for tailored treatment of patients. Current therapy options comprise supportive therapy, growth factor therapy, chemotherapy, immunotherapy, epigenetic therapy and allogeneic stem-cell transplantation (ASCT) [12, 13]. To date, ASCT is the only treatment considered as curative; however, due to high toxicity of the treatment and the advanced age of many MDS patients at diagnosis, it can only be applied on a limited subset of cases. Hypomethylating agents (HMAs) like 5-azacytidine (AZA) and decitabine have shown a high efficacy in MDS, especially in high-risk MDS patients, remaining the mainstay of treatment in this subtype of MDS, however only half of all patients will respond to these drugs [14–16]. The reasons underlying AZA resistance are unknown, and few alternatives exist for non-responders. Recently, it has been said that primary AZA resistance is intricately linked to cell cycle quiescence of hematopoietic progenitor cells (HPC) in non-responders before treatment, and AZA response is associated with the induction of an inflammatory response in HPCs *in vivo* [17]. Even so, there is a lack of biological markers that predict which patients will respond to HMAs, so more studies focusing in specific subtypes of MDS are needed.

In this study, we performed a mutation analysis of 83 genes involved in myeloid malignancies in a cohort of high-risk MDS and sAML patients treated uniformly with AZA according to a prospective multicenter protocol from the CETLAM Group. The aim of the study was to define the mutational status at diagnosis, to identify the relationship between genotype and treatment response, to establish if any gene mutation could be used as a prognostic marker for response and survival and to study the evolution of mutations during patients’ follow-up (response and relapse).

## RESULTS

### Patient characteristics and treatment given

A total of 39 patients with high-risk MDS and sAML were studied. Main clinical and biological characteristics of patients are summarized in Table 1. Median age at diagnosis was 71 years (range 55-83) and the series included 29 (74%) males and 10 (26%) females. According to the 2008 WHO classification cases were diagnosed of refractory anemia with ring sideroblasts (RARS) (*n =* 1), refractory cytopenia with multilineage dysplasia and ring sideroblasts (RCMD-RS) (*n =* 1), refractory cytopenia with multilineage dysplasia (RCMD) (*n =* 5, 13%), refractory anemia with excess of blasts-1 (RAEB-1) (*n =* 9, 23%), refractory anemia with excess of blasts-2 (RAEB-2) (*n =* 14, 36%) and sAML (*n =* 9, 23%). Patients risk stratification was made according to the Revised International Prognostic Scoring System (IPSS-R) score and all patients belonged to intermediate, high or very high-risk groups (Table 1). All patients were treated with AZA (at a dose of 75 mg/m^2^/d for 7 days, 5-2-2, every 4 weeks). The median number of AZA cycles was 6 (range 1–36).

**Table 1:** Main clinical and hematological characteristics of high-risk MDS and sAML patients at diagnosis (*n* = 39)

Variable	Median (range)	*N* = 39 (%)
Age, years	71 (55–83)	
<70 y		18 (46)
≥70 y		21 (54)
Gender		
Male		29 (74)
Female		10 (26)
WHO classification		
RARS		1 (2.5)
RCDM-RS		1 (2.5)
RCMD		5 (13)
RAEB-1		9 (23)
RAEB-2		14 (36)
sAML		9 (23)
Hemoglobin level, g/dL	9.1 (6.5–12.5)	
<10 g/dL		29 (74)
≥10 g/dL		10 (26)
Leukocyte count, × 10^9^/L	2.9 (1.1–50.2)	
<4 × 10^9^/L		29 (74)
>4 × 10^9^/L and <11 × 10^9^/L		8 (21)
≥11 × 10^9^/L		2 (5)
Platelet count, × 10^9^/L	63 (13–416)	
<100 × 10^9^/L		28 (72)
≥100 × 10^9^/L		11 (28)
Neutrophil count, × 10^9^/L	1.3 (0.09–13.55)	
<0.8 × 10^9^/L		13/36 (36)
≥0.8 × 10^9^/L		23/36 (64)
Blasts in PB, %	0 (0–20)	
<5%		32/38 (84)
≥5%		6/38 (16)
Blasts in BM, %	11 (0–36)	
<20%		29 (74)
≥20%		10 (26)
Cytogenetics		
Normal karyotype		8 (21)
Abnormal karyotype		31 (79)
IPSS risk group		
Intermediate-1		4 (10)
Intermediate-2		19 (49)
High	16 (41)	
IPSS-R risk group		
Intermediate		5 (13)
High		12 (31)
Very High		22 (56)

BM: bone marrow; PB: peripheral blood; RAEB-1: refractory anemia with excess of blasts-1; RAEB-2: refractory anemia with excess of blasts-2; RARS: refractory anemia with ring sideroblasts; RCMD: refractory cytopenia with multilineage dysplasia.

### Conventional cytogenetics

An informative result for conventional cytogenetics (CC) studies at diagnosis was obtained in all patients. Eight (21%) patients had a normal karyotype or a loss of chromosome Y. The rest of patients (79%) had an abnormal karyotype, being in most of cases a complex karyotype.

### Targeted deep sequencing

Targeted deep sequencing was performed in a total of 77 samples (39 at diagnosis, 17 follow-ups [6 at response and 11 at relapse] and 21 CD3+ control samples), with a mean depth per base per sample of 737-fold (range: 84-971). More than 95% of the target sequences were analyzed with >100 independent reads and >99% with at least 30 reads. After applying the mentioned filters in methodology, a mean of 2 variants per sample were called as high-probability somatic changes (range 0-5).

### Mutational analysis at diagnosis

**Figure 1:**
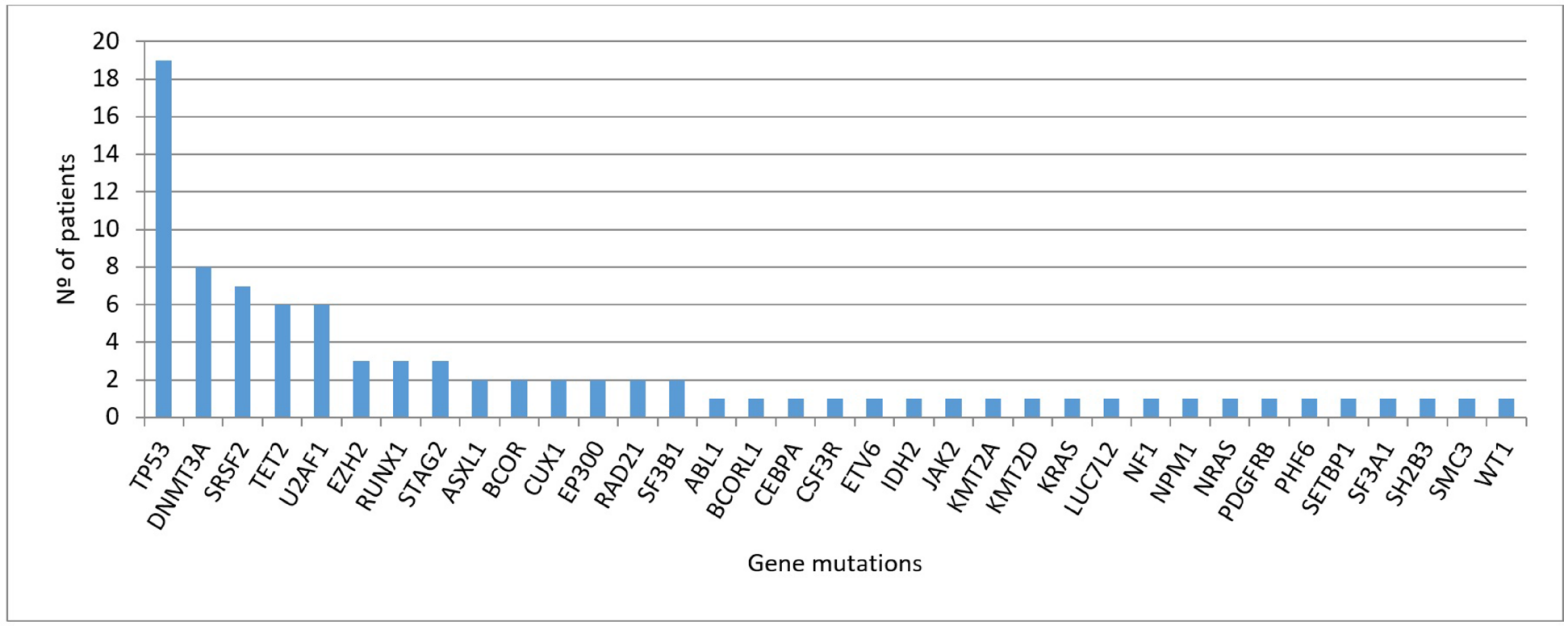
Distribution of mutations detected at diagnosis (*n* = 39 patients)

Across the entire cohort, 37/39 (95%) of patients harbored at least one mutation, affecting 35 of the 83 studied genes (in 48 genes we did not find any mutation). Details of all the detected variants are described in Supplementary Table 2. Due to the availability of the CD3+ control tissue, we were able to discard a mean of 2 variants per sample that had not been previously established as SNPs in public databases. Overall, the distribution of the number of mutations detected per patients was as follows, 12 (31%) patients had 1 mutation, 11 (28%) patients had 2 concurrent mutations, 2 (5%) patients had 3 mutations, 7 (18%) patients had 4 mutations and 5 (13%) patients had 5 mutations (Supplementary Figure 1A). The distribution of mutations at diagnosis across patients’ cohort is described in Figure 1. The most frequently affected genes (in >10% of patients) were *TP53* (49%), *DNMT3A* (21%) and *SRSF2* (18%); followed by *TET2* (15%) and *U2AF1* (15%). The list of frequencies of all the affected genes is described in Supplementary Table 3, and most of detected mutations corresponded to missense variants (Supplementary Figure 1B).

Regarding cytological category, the average of alterations detected in each category is detailed in Table 2, suggesting that patients with sAML present more variations than patients with RAEB-2, RAEB-1 or RCMD, even though this observation has to be taken with caution due to the number of patients in each category. We then examined the correlation between gene mutations to identify possible functional interactions across the different affected genes. Due to the heterogeneity of mutated genes in the cohort of patients, we focused the statistical analyses only in those mutations detected in at least five patients. The only association found was the correlation between *SRSF2* and *TET2* mutations (*P =* 0.006), and both mutations were mutually exclusive with *TP53* mutations (*P =* 0.008).

**Table 2:** Average of mutations per cytological category

	Number of patients	Average number of mutations per patient (range)
sAML	9	2.889 (1–5)
RAEB-2	14	2.286 (0–5)
RAEB-1	9	2 (1–4)
RCMD	5	2.2 (1–4)
RCMD-RS	1	5
RARS	1	1

RAEB-1: refractory anemia with excess of blasts-1; RAEB-2: refractory anemia with excess of blasts-2; RARS: refractory anemia with ring sideroblasts; RCMD: refractory cytopenia with multilineage dysplasia; RCMD-RS: refractory cytopenia with multilineage dysplasia and ring sideroblasts; sAML: secondary acute myeloid leukemia.

### Relations between gene mutations and clinical variables

We investigated the relation between mutations detected at diagnosis and main clinical and biological parameters of the patients, including age, gender, WHO subtypes, peripheral blood cell counts, percentage of blasts in peripheral blood and bone marrow, karyotype and IPSS-R categories. We observed more *SRSF2* mutations in patients with advanced age (>70 years), even though it wasn’t statistic significant (6 patients vs. 1 patient; *P =* 0.098), and these mutations were associated with the absence of a complex karyotype (*P =* 0.010). *U2AF1* mutations were associated with fewer neutrophils in peripheral blood (<0.8 × 10^9^/L) (*P =* 0.003). Patients with *TET2* wild-type were associated with less than 5% of blasts in peripheral blood (*P =* 0.021). Mutations in *TP53* gene were associated with age, younger than 70 years (*P =* 0.007), abnormal karyotype (*P =* 0.003), mainly with complex karyotype (*P <* 0.001), and with high-risk groups according to IPSS-R (*P =* 0.003).

We also studied the effect of *TP53* mutations in the subset of patients that underwent ASCT (*n =* 5); three of them harbored a *TP53* mutation and died in less than one year from MDS diagnosis. The other two patients, without *TP53* mutations, remain alive after more than 3 years after diagnosis.

### Overall survival and progression free survival analysis

**Figure 2:**
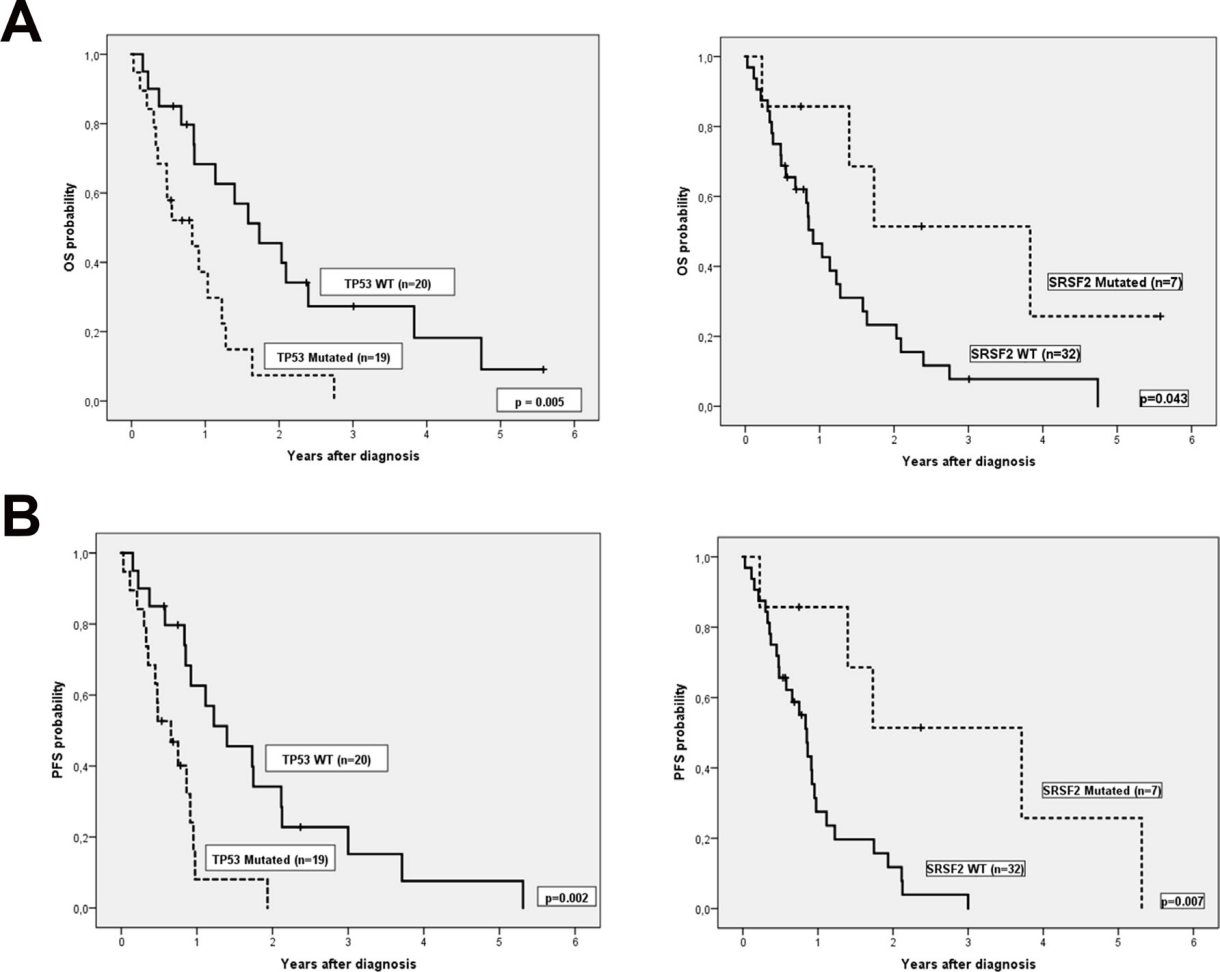
(**A**) Overall survival and (**B**) Progression free survival according TP53 and SRSF2 mutational status.

**Figure 3:**
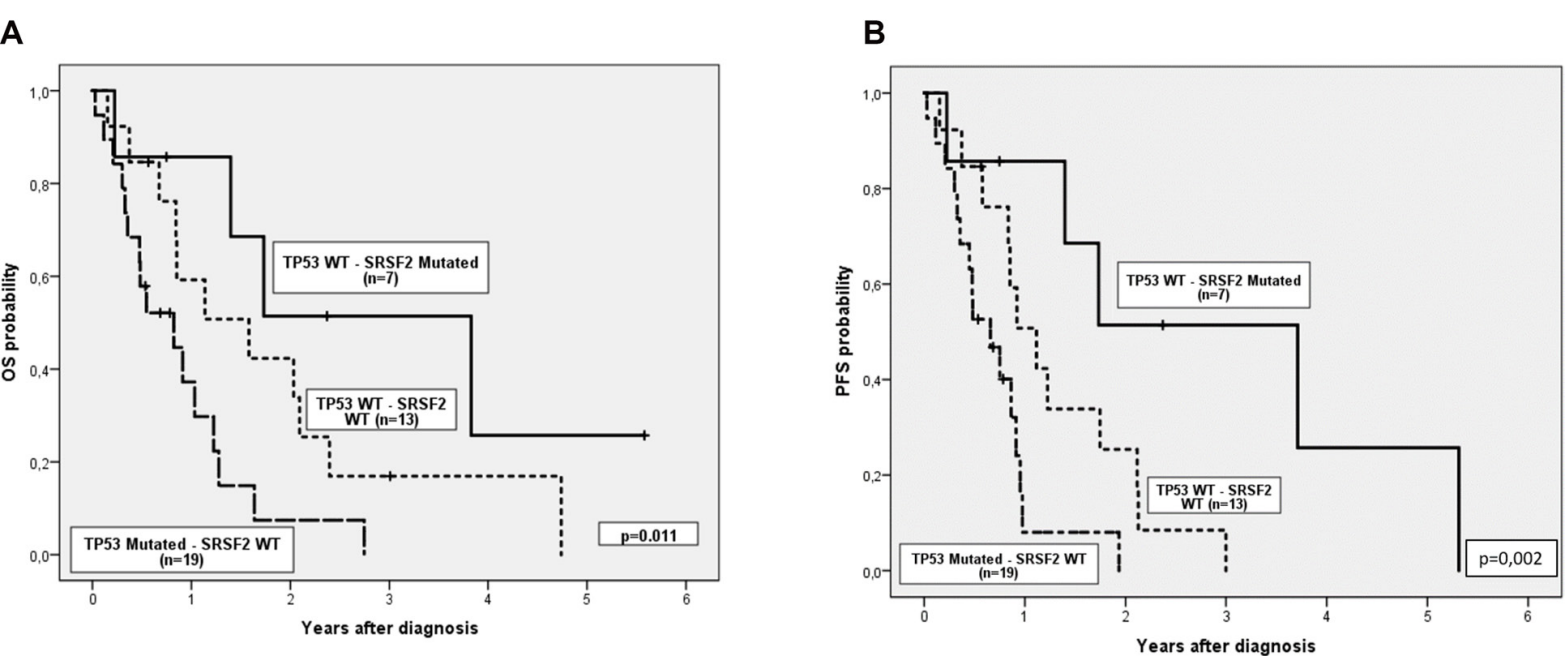
(**A**) Overall survival and (**B**) Progression free survival considering together TP53 and SRSF2 mutational status.

We then explored the impact of clinical and biological data on patients’ outcome. Median follow-up of alive patients was 11 months (range 0–68), and median OS and PFS (95% CI) of the cohort were 1.1 years (0.6, 1.6) and 0.9 years (0.8, 1), respectively. Complex karyotype, IPSS-R risk group and hemoglobin were predictive of both OS and PFS (Table 3). In addition, there was a trend to worse PFS in patients with more than 5% of blasts in peripheral blood at diagnosis. Regarding genetic features, we did not find any association between the number of mutations and OS or PFS. Focusing on specifics genes, only mutations in *TP53* were associated with shorter OS and PFS, while mutations in *SRSF2* correlated with better OS and PFS (Figure 2). Considering together *SRSF2* and *TP53* mutational status, patients could be stratified in three groups with significant different rates of OS and PFS (Figure 3).

**Table 3:** Results of overall survival and progression free survival univariate and multivariate analyses

UNIVARIATE ANALYSIS					
Variable	Categories	Overall Survival (OS)		Progression Free Survival (PFS)	
		Median OS (95% CI)	*P* value	Median PFS (95% CI)	*P* value
Karyotype	Normal	2.1 (1.2, 3)	0.022	1.75 (1.7, 1.8)	0.01
	Altered	0.9 (0.7, 1)		0.8 (0.6, 1.1)	
Hemoglobin level	<100	0.9 (0.5, 1.2)	0.028	0.8 (0.5, 1.1)	0.029
	≥100	2.1 (0.6, 3.6)		1.7 (0.3, 3.1)	
IPSS-R	Intermediate	2 (0, 4.6)	0.026	1.2 (0.2, 2.2)	0.005
	High	1.7 (1.2, 2.2)		1.7 (0.8, 2.7)	
	Very high	0.5 (0.1, 1)		0.5 (0.2, 0.8)	
*TP53*	WT	1.7 (0.9, 2.6)	0.005	1.4 (0.6, 2.2)	0.002
	Mutated	0.8 (0.2, 1.4)		0.7 (0.3, 1)	
*SRSF2*	WT	0.9 (0.7, 1.2)	0.043	0.9 (0.7, 1)	0.007
	Mutated	3.8 (1.5, 6.1)		3.7 (1.5, 5.9)	

MULTIVARIATE ANALYSIS					
Variable	Baseline category	Overall Survival (OS)^*^		Progression Free Survival (PFS)^**^	
		HR (95% CI)	*P* value	HR (95% CI)	*P* value
*TP53*	WT	2.9 (1.3, 6.3)	0.007	3.4 (1.5, 7.5)	0.003

Finally, we performed an adjusted multivariable Cox model including cytogenetic, IPSS-R and *TP53* mutational status (Table 3). For both OS and PFS, *TP53* mutational status was the only variable which remained significant.

### Predicting factors of response to hypomethylating therapy

In our cohort of patients, 16 cases were classified as responders (8 CR and 8 PR) to AZA at six months and 8 cases were classified as non-responders (2 stable disease and 6 treatment failure). There were 15 patients who died before the sixth cycle, but from those patients who received a minimum of 3 cycles of AZA we assessed their response according to the percentage of blasts in bone marrow and their transfusion requirements during these months. Afterwards, we could incorporate into the analysis seven more patients, six as non-responders, because their transfusions requirements did not decrease or because the blasts percentage increased under AZA treatment, and one patient was considered a responder because his transfusion requirements decreased with AZA treatment before being allografted. The other 8 patients received less than 3 AZA cycles and they were considered as no evaluable for response predicting factors.

The overall response rate (ORR) for evaluable patients was 55% (17/31). Univariate analysis of the impact of clinical characteristics and mutational status on response rates are summarized in Table 4. We did not find any clinical variable or gene mutation associated with treatment response.

**Table 4:** Differences in treatment response based on clinical characteristics and mutational status from 31 evaluable patients

Variable	Non-responders *n* = 14 (%)	Responders *n* = 17(%)	*p*-value
Age, years			0.524
<70	5 (36)	8 (47)	
≥70	9 (64)	9 (53)	
Gender			0.412
Male	9 (64)	14 (82)	
Female	5 (36)	3 (18)	
WHO classification			-
RARS	1 (7)	0	
RCDM-RS	1 (7)	0	
RCMD	1 (7)	4 (24)	
RAEB-1	5 (36)	1 (6)	
RAEB-2	4 (29)	7 (41)	
sAML	2 (14)	5 (29)	
Hemoglobin level, g/dL			0.132
<10 g/dL	12 (86)	10 (59)	
≥10 g/dL	2 (14)	7 (41)	
Leukocyte count, × 10^9^/L			0.112
<4 × 10^9^/L	8 (57)	14 (82)	
>4 × 10^9^/L and <11 × 10^9^/L	6 (43)	2 (12)	
≥11 × 10^9^/L	0	1 (6)	
Platelet count, × 10^9^/L			1
<100 × 10^9^/L	10 (71)	12 (71)	
≥100 × 10^9^/L	4 (29)	5 (29)	
Neutrophil count, × 10^9^/L			0.177
<0.8 × 10^9^/L	3 (23)	8 (47)	
≥0.8 × 10^9^/L	10 (77)	9 (53)	
Blasts in PB, %			0.157
<5%	10 (71)	15 (94)	
≥5%	4 (29)	1 (6)	
Blasts in BM, %			0.240
<20%	12 (86)	11 (65)	
≥20%	2 (14)	6 (35)	
Cytogenetics			1
Normal karyotype	3 (21)	4 (24)	
Abnormal karyotype	11 (79)	13 (76)	
IPSS risk group			0.816
Intermediate-1	2 (14)	2 (12)	
Intermediate-2	7 (50)	7 (41)	
High	5 (36)	8 (47)	
IPSS-R risk group			0.739
Intermediate	3 (21)	2 (12)	
High	5 (36)	6 (35)	
Very High	6 (43)	9 (53)	
*TP53* mutational status			0.925
Wild-type	8 (57)	10 (59)	
Mutant	6 (43)	7 (41)	
*DNMT3A* mutational status			0.698
Wild-type	11 (79)	12 (71)	
Mutant	3 (21)	5 (29)	
*SRSF2* mutational status			1
Wild-type	11 (79)	14 (82)	
Mutant	3 (21)	3 (18)	
*TET2* mutational status			0.636
Wild-type	11 (79)	15 (88)	
Mutant	3 (21)	2 (12)	
*U2AF1* mutational status			0.232
Wild-type	14 (100)	14 (82)	
Mutant	0	3 (18)	

BM: bone marrow; PB: peripheral blood; RAEB-1: refractory anemia with excess of blasts-1; RAEB-2: refractory anemia with excess of blasts-2; RARS: refractory anemia with ring sideroblasts; RCMD: refractory cytopenia with multilineage dysplasia; RCMD-RS: refractory cytopenia with multilineage dysplasia and ring sideroblasts; sAML: secondary acute myeloid leukemia.

### Mutational follow-up during response, relapse or AML progression

**Figure 4:**
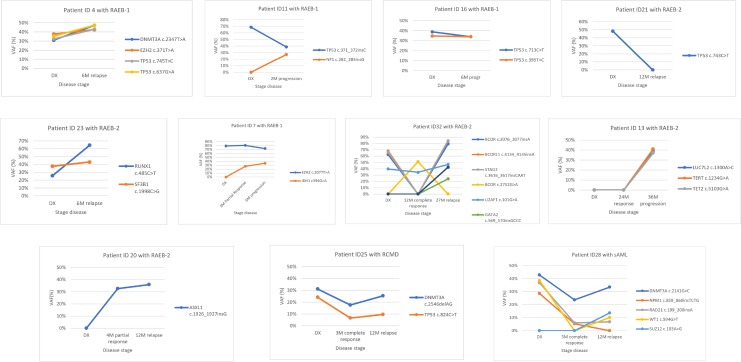
Variant allele frequency evolution during disease follow-up in eleven patients treated with 5-azacytidine In 5 patients (ID4, ID11, ID16, ID21 and ID23) samples were available at diagnosis (DX) and relapse/progression. In 6 patients (ID7, ID13, ID20, ID25, ID28 and ID32) samples were available at diagnosis, response and relapse/progression. In both cases different patterns can be seen: Model 1: The variant allele frequency (VAF) of mutations, or the number of mutations, decreases on response and increases at progression (patient ID4, ID11, ID13, ID23, ID 25, ID28 and ID32). Model 2: Mutation’s VAF, or the number of mutations, increases on response indicating the progression of the disease even though an improvement of hematological features (patient ID7 and ID20) or no change has been seen between diagnosis and progression (ID16). Model 3: Mutation’s VAF, or the number of mutations, decreases or disappears on disease progression indicating that the cause of progression is not due to the mutations found at diagnosis (patient ID21).

Targeted deep sequencing was performed at response in six patients and at relapse or AML transformation in eleven patients (Table 5). In two patients the number of mutations at response decreased (patients ID32 and ID28), two patients maintained the number of mutations (patients ID13 and ID25) and in two more patients the number of mutations increased (patients ID7 and ID20), even though they had hematological improvement (Figure 4). The number of mutations detected per patient was the same between diagnosis and progression in 4/11 patients (ID4, ID16, ID23 and ID25) (36.4%) and increased at time of relapse or AML progression in 6/11 patients (ID7, ID11, ID13, ID20, ID28 and ID32) (54.5%). In patient ID32, the dominant clone observed at diagnosis disappeared in complete response but reappeared on relapse with mutations in other genes. Only one patient (ID21) did not present at relapse the mutation found at diagnosis or any other mutation from the studied gene panel.

**Table 5:** List of affected genes in MDS patients that were studied at diagnosis, response and/or at time of progression/relapse (*n* = 11)

Data at diagnosis			Data at response			Data at progression/ relapse		
Pt ID and DX	Alteration	VAF	Time	Alteration	VAF	Time	Alteration	VAF
Pt ID4	DNMT3A c.2347T>A	31%		^*^		6M	DNMT3A c.2347T>A	47.1%
RAEB-1	EZH2 c.371T>A	37.6%				relapse	EZH2 c.371T>A	42.5%
	TP53 c.745T>C	32.3%					TP53 c.745T>C	42.9%
	TP53 c.637G>A	35.3%					TP53 c.637G>A	47.2%
Pt ID16	TP53 c.713C>T	38.8%				6M	TP53 c.713C>T	34%
RAEB-1	TP53 c.395T>C	34.7%				progression	TP53 c.395T>C	34%
Pt ID7	EZH2 c.2077T>A	79.4%	3M	EZH2 c.2077T>A	80.7%	6M	EZH2 c.2077T>A	72.6%
RAEB-1			Partial response	**IDH1 c394G>A**	27.4%	progression	**IDH1 c394G>A**	35.3%
Pt ID11	TP53 c.371_372insC	68.6%		^*^		2M	TP53 c.371_372insC	38.8%
RAEB-1						progression	**NF1 c.282_283insG**	27%
Pt ID32	BCOR c.2076_2077insA	62.4%	12M	**BCOR c.2752G>A**	51.2%	27M	BCOR c.2076_2077insA	79.3%
RAEB-2	BCORL1 c.4134_4135insA	68.2%	Complete	U2AF1 c.101G>A	34.6%	Relapse	BCORL1 c.4134_4135insA	84.6%
	STAG2 c.3616_3617insCAAT	65.8%	response				STAG2 c.3616_3617insCAAT	84.1%
	U2AF1 c.101G>A	39.7%					U2AF1 c.101G>A	46.8%
							**GATA2 c.569_570insGCCC**	24.1%
							**NF1 c.1067T>C**	42.6%
Pt ID21	TP53 c.743C>T	48.2%		^*^		12M	0	
RAEB-2						relapse		
Pt ID13	0		24M	0		36M	**LUC7L2 c.1300A>C**	38.9%
RAEB-2			response			progression	**TERT c.1234G>A**	40.9%
							**TET2 c.5103G>A**	37%
Pt ID20	0		4M	**ASXL1 c.1926_1927insG**	32.6%	12M	**ASXL1 c.1926_1927insG**	35.9%
RAEB-2			Partial response			relapse		
Pt ID23	RUNX1 c.485C>T	25.4%		^*^		6M	RUNX1 c.485C>T	64.3%
RAEB-2	SF3B1 c.1998C>G	37.6%				Relapse	SF3B1 c.1998C>G	43.1%
Pt ID25	DNMT3A c.2546delAG	31.13%	3M	DNMT3A c.2546delAG	17.6%	12M	DNMT3A c.2546delAG	25.3%
RCMD	TP53 c.824C>T	24.08%	Complete	TP53 c.824C>T	6.6%	relapse	TP53 c.824C>T	9.6%
			response					
Pt ID28	DNMT3A c.2141G>C	42.8%	5M	DNMT3A c.2141G>C	23.7%	12M	DNMT3A c.2141G>C	33.5%
sAML	NPM1 c.859_860insTCTG	28.6%	Complete	NPM1 c.859_860insTCTG	5.25%	relapse	RAD21 c.199_200insA	6.8%
	RAD21 c.199_200insA	36.8%	response	RAD21 c.199_200insA	5.96%		WT1 c.594G>T	10.1%
	WT1 c.594G>T	38.5%					**SUZ12 c.103A>G**	13.6%

DX: diagnosis; M: month; Pt: patient; RAEB-1: refractory anemia with excess of blasts-1; RAEB-2: refractory anemia with excess of blasts-2; RCMD: refractory cytopenia with multilineage dysplasia; sAML: secondary acute myeloid leukemia; VAF: Variant allele frequency.

^*^ No data at that point.

Genes that differ from the affected at time of diagnosis are highlighted in bold.

## DISCUSSION

MDS is a heterogeneous disease, and so are the genetic alterations more appellants, as they include gains or losses of chromosomal regions, mutations and epigenetic modifications [18–20]. Over the past few years, next-generation sequencing (NGS) has led to a revolution in the study of hematological malignancies, with remarkable efforts to characterize the mutational basis of these disorders. Focusing on MDS, targeted deep-sequencing has identified a landscape of mutated genes that encode signal transduction proteins (*NRAS , FLT3-ITD, CBL, JAK2, KIT*), transcription factors (*RUNX1, ETV6*), tumor suppressors genes (*TP53, WT1*), epigenetic modifiers (*TET2, ASXL1, IDH1, IDH2, EZH2, DNMT3A*), RNA splicing machinery (*SF3B1, U2AF1, SRSF2, ZRSR2*) and components of the cohesin complex (*STAG2, RAD21, SMC3, SMC1A*) [10, 18, 21]. However, no mutations in these genes are detected in 10–20% of MDS patients. In our series, using a panel of 83 myeloid related genes, we were able to detect mutations in 95% of patients with high-risk MDS and sAML. This percentage is higher than other studies focused on MDS, maybe due to our patients are high-risk and this supports the idea that in advanced stages of the disease there is a greater genomic complexity. Only 5 genes (*TP53*, *DNMT3A, SRSF2, TET2* and *U2AF1*) were present in more than 10% of cases. We found mutations in 30 additional genes, but in a lower frequency (Figure 1) and no mutation was found in the rest of the studied genes (*n =* 48). These results corroborate the heterogeneous landscape of MDS at the mutational level. We would also like to emphasize, that many groups do not use control tissue when NGS is performed, may be due to the increase on cost that this implies, but we have demonstrated its usefulness to discriminate germ line variations from somatic mutations. In our study, due to the availability of the CD3+ control tissue, we have been able to discard a mean of two variables per sample that had not been established as SNPs in public databases. Otherwise it would have wrongly increased the number of mutations detected per patient.

In MDS the percentage of *TP53* mutations is approximately 20% [13] but in our cohort this percentage increased up to nearly 50%. This fact could be explained because our cohort of patients include only high-risk MDS and sAML, which have been highly associated with complex karyotypes (46% in our series) and it is also well known that *TP53* mutations are enriched in patients with these cytogenetic characteristics [22]. Another explanation could be that the limited number of patients included in our series have influenced this percentage. Recently, some studies have demonstrated the association between mutations in *TP53* and higher response rate to high doses of decitabine in AML and MDS patients [23, 24]. Although AZA and decitabine are both HMAs, we could not corroborate these results in our cohort treated with AZA. An explanation may be the different treatment schedules or doses used between the two drugs, as well as differences mechanisms of action of both treatments (incorporation into DNA of decitabine and incorporation into RNA and DNA of AZA) [25], or even though to specific disease characteristics of the patients treated within each study.

Due to the large amount of genetic information currently available, some groups are working in incorporating molecular data into the IPSS-R in patients with MDS [26]. As *TP53* mutations have recurrently been associated with decreased OS and higher rates of AML transformation [7, 11], it seems to be a good candidate for being incorporated to this score. We have also corroborated this finding in our study, as *TP53* mutations were the only marker that retained its significance in the multivariate analysis for PFS and almost for OS. Mutations in *TP53* are also associated with poorer OS after ASCT [27, 28]. In accordance to these studies, and although in our series only 5 patients were allografted, the three patients who had *TP53* mutations died in less than one year after the MDS diagnosis, whereas the two patients without *TP53* alterations are still alive after more than 3 years after diagnosis.

It is well established that spliceosome mutations, such as *SRSF2*, are mutually exclusive among them but they usually coexist with mutations in epigenetic modifiers, cooperating to give rise to the MDS phenotype [29]. In our cohort we also observed this association between *SRSF2* and *TET2*. The role of *SRSF2* mutations in MDS is not yet well understood; while in some studies, these mutations have been associated with worse OS and a higher rate of transformation to AML [30], other groups have demonstrated that *SRSF2* mutations do not have impact on OS [31]. In our cohort, *SRSF2* mutations was an independent variable for better OS and PFS in the univariate analysis and together with *TP53* mutations it allows to stratify patients into three risk group categories (Figure 3). These results have to be taken with caution as in our series, *SRSF2* mutations were associated with *TET2* mutations (4/7) (which have been defined as a factor of response to HMAs [32] particularly when *ASXL1* is not mutated) and none of these four patients had *ASXL1* mutations. Considering also the limited number of patients with *SRSF2* mutations in our study, the good prognosis of *SRSF2* mutations should be confirmed in a bigger independent cohort of high-risk MDS patients.

There are some studies that have shown a relationship between a higher number of oncogenic mutations with an adverse outcome [20]. In our cohort we could not demonstrate this relationship (data not shown) maybe due to the high proportion of patients with *TP53* mutations, which by itself gives a worse outcome to the patient.

Even though there are some studies that correlate mutational status to treatment response [23, 24, 32] we could not demonstrate this impact in our cohort. This fact could be explained by the limited number of evaluable patients for treatment response or due to differences between the designs of the studies.

Regarding MDS architecture, it has been defined that most cases of MDS are clonally heterogeneous, with a founding clone and multiple additional subclones. Using targeted deep sequencing techniques, it has become possible to find driver mutations involved in clonal evolution of MDS [10, 20, 33]. Driver mutations are defined as mutations in an immature hematopoietic stem cell with capacity for self-renewal; typically involving a gene of RNA splicing or DNA methylation, that provides selective advantage and determines local clonal expansion [7, 20]. To elucidate differential roles of mutations in MDS, we investigated clonal dynamics using targeted deep sequencing in 11 patients. Most of the mutations that we found at diagnosis were in genes that control cell cycle (*TP53*), DNA methylation (*DNMT3A*) and spliceosome machinery (*SRSF2*). The mean number of gene mutations in a patient with MDS tends to be higher in the high-risk subtypes, supporting the idea that some MDS stem cells gain the ability to proliferate through the accumulation of gene mutations, leading to clonal expansion and disease progression [10]. At progression, most of patients from our cohort experienced an increase in the number of mutations, their diversity and clone sizes, with alterations frequently found in the dominant clones with or without their sweeping previous clones. It has been described that the emergence of new driver mutations, even if they are still subclonal, can have relevant implications for the future disease evolution. In our cohort we could observe two patients with an increase in the number of mutations despite having hematological improvement, and these patients progressed faster than the other ones. Studying mutation evolution during patient’s follow-up may allow to identify patients whose disease will progress faster, even before symptoms appear.

In summary, although we are aware that, compared with other studies, our series is limited by the number of studied samples, we have one of the better well studied cohorts of a homogeneous subtype of MDS, in which DNA from CD3+ cells as control tissue was available to avoid germinal variables. Our findings corroborate the higher incidence of *TP53* mutations among high-risk MDS, that molecular markers such as *TP53* and *SRSF2* mutations provide additional prognostic data to guide clinical decisions in high-risk MDS and that the use of mutation analysis during follow up may help to identify patients who are progressing before the onset of signs of progression. All these facts emphasize the importance of introducing genetic data into prognostic models to better stratify patients at diagnosis. Nonetheless, future studies including larger cohorts of patients treated homogenously with AZA are needed to consolidate our results.

## MATERIALS AND METHODS

### Patients and samples

A total of 39 high-risk MDS (*n =* 30) and sAML (*n =* 9) patients, at diagnosis and after AZA treatment, were retrospectively analyzed in this study. Samples were collected from October 2009 to December 2014. Patients were diagnosed according to the 2008 World Health Organization (WHO) classification [34] in different hospitals from the CETLAM Group, and were treated uniformly with AZA (75 mg/m^2^ per day for 7 days, 5-2-2, every 4 weeks). All patients received AZA during their disease evolution and five patients were allografted after AZA treatment. Response to treatment was assessed using the International Working Group (IWG) Response Criteria [35]. Briefly, patients in a complete response (CR), partial response (PR), marrow CR (mCR) or hematologic improvement (HI) after six months of treatment were considered as “responders”, while patients who had stable disease, no response or disease progression were considered as “non-responders”. Study approval was obtained from Ethical Committee for Clinical Research from Hospital Germans Trias i Pujol. Informed consent was given by all patients, in accordance with the Declaration of Helsinki.

### Cytogenetics

Conventional G-banding cytogenetics was performed on bone marrow samples at diagnosis in each center and karyotypes were described according to the International System for Human Cytogenetic Nomenclature 2013 [36].

### DNA samples

Samples were collected at diagnosis for all 39 patients and at time of response and/or progression in 11 patients, all these samples were sent to a reference laboratory. Whole bone marrow samples were used for the targeted deep sequencing analysis. In 21 out of 39 patients, peripheral CD3+ T lymphocytes were purified with immunomagnetic separation (Miltenyi Biotec, Bergisch Gladbach, Germany) according to the manufacturer’s recommendations and were used as control tissue to discriminate germ line variations from somatic mutations. Genomic DNA was extracted with QiaAmp DNA Blood Mini kit (Qiagen, Hilden, Germany) and quantified using Quant-iT PicoGreen dsDNA Assay Kit (Invitrogen, CA, USA).

### Targeted deep sequencing

Targeted deep-sequencing of a panel of 83 myeloid-related genes was performed in all samples (Supplementary Table 1). Indexed libraries were prepared with 1 μg of double strand genomic DNA using the Kapa Library Preparation Kit (Kapa Biosystems, MA, USA). Custom target capture enrichment using the SeqCap EZ capture chemistry (Nimblegen, Roche, Basel, Switzerland) was performed on pools of 8 libraries. Multiplexed captured libraries were sequenced on an Illumina MiSeq following a 150bp paired-end reads standard protocol.

### Targeted sequencing data analysis

Sequencing data were analyzed using the commercial softwares MiSeq Reporter and Variant Studio (Illumina, CA, USA). High-probability oncogenic mutations were called after eliminating sequencing and mapping errors and after discarding variants located in high variable regions, with low coverage (<20 reads of the variant) or with a variant allele frequency (VAF) <10% for single nucleotide variants (SNVs) and <15% for insertions and deletions (indels) to ensure they were not technical artefacts. VAF was calculated as the number of the variant reads divided by the total number of reads for that position. Single nucleotide polymorphisms (SNPs) described on human genetic variation databases were also excluded.

The information obtained from patient-specific control tissue was also used to confirm SNPs filtering strategy when available. The remaining variants were considered as candidate somatic mutations, and were finally tagged as oncogenic, based on the information derived from the literature and/or on the information given by *in silico* predictors (data are available in SRA with SRP133179 reference).

### Statistical analysis

Baseline characteristics were described as frequency and percentage for categorical variables and median and range for quantitative variables. Comparisons of categorical variables between patient subsets were compared using χ^*2*^ or Fisher’s exact test, when appropriate, while median test was used to compare continuous variables. OS was defined as time from diagnosis to the last follow-up or death from any cause and progression free survival (PFS) as time from diagnosis to progression or death related to disease [35]. Patients who underwent an ASCT were censored at that time for OS and PFS analysis. Survival curves were performed using the Kaplan-Meier method and log-rank test was used for comparisons between groups. Multivariate analysis was performed using Cox proportional-hazards regression model, considering Wald Backward as selection method. Two-sided *P* values < 0.05 were considered as statistically significant. The statistical package SPSS, version 24.0 (SPSS Inc., Chicago, IL, USA) was used for all analyses.

## SUPPLEMENTARY MATERIALS FIGURE AND TABLES




