# Supplementary material for: Impact of mutational studies on the diagnosis and the outcome of high-risk myelodysplastic syndromes and secondary acute myeloid leukemia patients treated with 5-azacytidine
Source: Oncotarget. 2018 Apr 10;9(27):19342–55. doi: 10.18632/oncotarget.25046 (PMC5922401; doi:10.18632/oncotarget.25046)
Supplement: Supplementary file 1 [file oncotarget-09-19342-s001.pdf]

Impact of mutational studies on the diagnosis and the outcome of high–risk myelodysplastic syndromes and secondary acute myeloid leukemia patients treated with 5–azacytidine

SUPPLEMENTARY MATERIALS

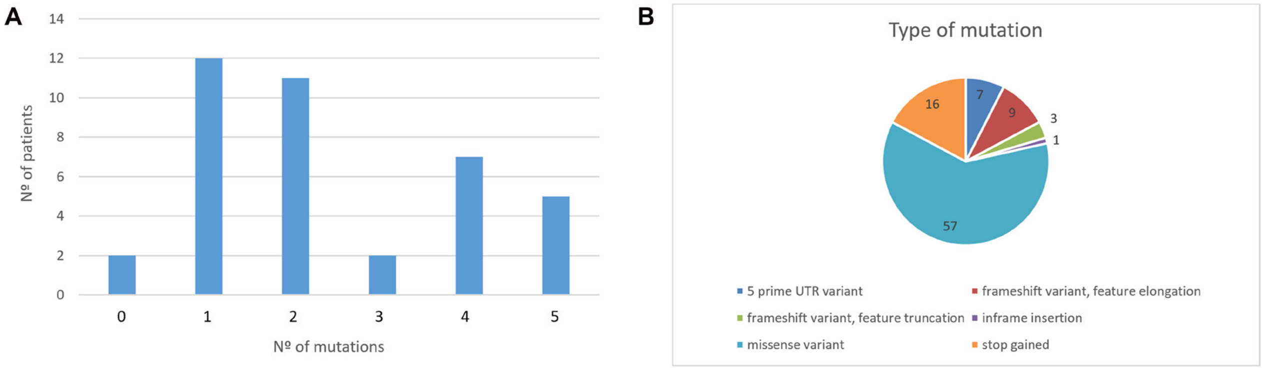

Supplementary Figure 1: Number and type of mutations across the high-risk MDS and sAML patients at diagnosis. (**A**) Distribution of number of mutations detected per patient; (**B**) type of mutation.

Supplementary Table 1: Genes included in the 83 myeloid–related gene panel

Gene	Target region (exon)	Gene	Target region (exon)	Gene	Target region (exon)	Gene	Target region (exon)
ABL1	4–9	*EED*	full	*MECOM*	full	*SF3B1*	10–16
AEBP2	full	*EP300*	full	*KMT2A*	full	*SFPQ*	full
ASXL1	9, 11, 12	*ETV6*	full	*MLL2*	full	*SH2B3*	full
ATRX	full	*EZH2*	full	*MPL*	10	*SMC1A*	full
BCOR	full	*FLT3*	14, 15, 20	*NF1*	full	*SMC3*	full
BCORL1	full	*GATA1*	2	*NPM1*	11, 12	*SPARC*	full
BRAF	full	*GATA2*	full	*NRAS*	1–3	*SRSF2*	1
CALR	9	*GCAT*	full	*PDGFRA*	full	*STAG1*	full
CBL	8, 9	*GNAS*	full	*PDGFRB*	full	*STAG2*	full
CBLB	9, 10	*HRAS*	2, 3	*PHF6*	full	*SUZ12*	full
CDH13	full	*IDH1*	4	*PHLPP1*	full	*TERC*	full
CDH3	full	*IDH2*	4	*PTEN*	5–8	*TERT*	full
CDKN2A	full	*IKZF1*	full	*PTPN11*	full	*TET2*	2–11
CEBPA	full	*IRF1*	full	*RAD21*	full	*TGM2*	full
CREBBP	full	*JAK2*	12–16	*RPS14*	full	*TIMM50*	full
CSF3R	full	*JAK3*	13	*RUNX1*	3–8	*TP53*	4–11
CSNK1A1	full	*JARID2*	full	*SALL4*	full	*U2AF1*	2, 6
CTCF	full	*KDM6A*	full	*SBDS*	full	*UMODL1*	full
CTNNA1	full	*KIT*	2, 8–11, 13, 17	*SETBP1*	4	*WT1*	7, 9
CUX1	full	*KRAS*	1–3	*SF1*	full	*ZRSR2*	full
DNMT3A	full	*LUC7L2*	full	*SF3A1*	full		

Supplementary Table 2: Detected variants in the whole cohort of high–risk MDS and sAML patients at diagnosis (*n* = 39). See Supplementary_Table _2

Supplementary Table 3: Frequencies of the affected genes in the whole cohort of high-risk MDS and sAML patients at diagnosis (*n* = 39)

Gene	Nº variations	Nº patients	% patients that have alterations in this gene
TP53	24	19	48,72%
DNMT3A	8	8	20,51%
SRSF2	7	7	17,95%
TET2	6	6	15,38%
U2AF1	6	6	15,38%
EZH2	3	3	7,69%
RUNX1	3	3	7,69%
STAG2	3	3	7,69%
RAD21	2	2	5,13%
ASXL1	2	2	5,13%
BCOR	2	2	5,13%
CUX1	2	2	5,13%
EP300	2	2	5,13%
SF3B1	2	2	5,13%
ABL1	1	1	2,56%
BCORL1	1	1	2,56%
CEBPA	1	1	2,56%
CSF3R	1	1	2,56%
ETV6	1	1	2,56%
IDH2	1	1	2,56%
JAK2	1	1	2,56%
KMT2A	1	1	2,56%
KMT2D	1	1	2,56%
KRAS	1	1	2,56%
LUC7L2	1	1	2,56%
NF1	1	1	2,56%
NPM1	1	1	2,56%
NRAS	1	1	2,56%
PDGFRB	1	1	2,56%
PHF6	1	1	2,56%
SETBP1	1	1	2,56%
SF3A1	1	1	2,56%
SH2B3	1	1	2,56%
SMC3	1	1	2,56%
WT1	1	1	2,56%
