# Supplementary material for: Impact of mutational studies on the diagnosis and the outcome of high-risk myelodysplastic syndromes and secondary acute myeloid leukemia patients treated with 5-azacytidine
Source: Oncotarget. 2018 Apr 10;9(27):19342–55. doi: 10.18632/oncotarget.25046 (PMC5922401; doi:10.18632/oncotarget.25046)
Supplement: Supplementary file 2 [file oncotarget-09-19342-s002.docx]

**Supplementary Table 2**. Detected variants in the whole cohort of high-risk MDS and sAML patients at diagnosis (n=39)

| **Patient**  **ID** | **Gene** | **Variant** | **Chr** | **Coordinate (hg19)** | **Type** | **VAF (%)** | **Consequence** |
| --- | --- | --- | --- | --- | --- | --- | --- |
| 29 | *ABL1* | G>G/A | 9 | 133738189 | snv | 51,26 | missense_variant |
| 33 | *ASXL1* | CG>CG/C | 20 | 31022642 | deletion | 37,52 | frameshift_variant, feature_truncation |
| 29 | *ASXL1* | C>C/G | 20 | 31022288 | snv | 43,13 | stop_gained |
| 32 | *BCOR* | G>G/GA | X | 39932522 | insertion | 62,41 | frameshift_variant, feature_elongation |
| 14 | *BCOR* | G>G/C | X | 39921510 | snv | 85,84 | stop_gained |
| 32 | *BCORL1* | C>C/CA | X | 129162665 | insertion | 68,23 | frameshift_variant, feature_elongation |
| 30 | *CEBPA* | G>G/GGCGGGT | 19 | 33792731 | insertion | 28,22 | inframe_insertion |
| 37 | *CSF3R* | C>C/T | 1 | 36935408 | snv | 52,09 | missense_variant |
| 39 | *CUX1* | G>G/T | 7 | 101882800 | snv | 28,12 | stop_gained |
| 17 | *CUX1* | G>G/A | 7 | 101758502 | snv | 35,9 | missense_variant |
| 22 | *DNMT3A* | G>G/A | 2 | 25467083 | snv | 30,74 | stop_gained |
| 4 | *DNMT3A* | T>T/A | 2 | 25462060 | snv | 31,02 | stop_gained |
| 25 | *DNMT3A* | CAG>CAG/C | 2 | 25458625 | deletion | 31,13 | frameshift_variant, feature_truncation |
| 3 | *DNMT3A* | G>G/A | 2 | 25457243 | snv | 41,81 | missense_variant |
| 5 | *DNMT3A* | G>G/A | 2 | 25463248 | snv | 41,87 | missense_variant |
| 28 | *DNMT3A* | G>G/C | 2 | 25463541 | snv | 42,77 | missense_variant |
| 14 | *DNMT3A* | A>A/C | 2 | 25463229 | snv | 42,87 | missense_variant |
| 39 | *DNMT3A* | G>G/A | 2 | 25463184 | snv | 44,61 | missense_variant |
| 3 | *EP300* | C>C/A | 22 | 41547994 | snv | 45,43 | missense_variant |
| 37 | *EP300* | C>C/T | 22 | 41546029 | snv | 57,77 | missense_variant |
| 30 | *ETV6* | C>C/CA | 12 | 12038928 | insertion | 22,56 | frameshift_variant, feature_elongation |
| 4 | *EZH2* | T>T/A | 7 | 148526933 | snv | 37,58 | missense_variant |
| 7 | *EZH2* | T>T/A | 7 | 148506435 | snv | 79,4 | missense_variant |
| 19 | *EZH2* | T>T/C | 7 | 148526897 | snv | 90,11 | missense_variant |
| 33 | *IDH2* | C>C/T | 15 | 90631934 | snv | 47,22 | missense_variant |
| 37 | *JAK2* | G>G/T | 9 | 5073770 | snv | 14,29 | missense_variant |
| 27 | *KMT2A* | C>C/A | 11 | 118374121 | snv | 49,34 | missense_variant |
| 31 | *KMT2D* | C>C/T | 12 | 49424704 | snv | 36,25 | missense_variant |
| 12 | *KRAS* | C>C/T | 12 | 25380283 | snv | 41,94 | missense_variant |
| 24 | *LUC7L2* | G>G/C | 7 | 139091978 | snv | 25,5 | missense_variant |
| 17 | *NF1* | G>G/A | 17 | 29684020 | snv | 28,79 | missense_variant |
| 28 | *NPM1* | C>C/CTCTG | 5 | 170837543 | insertion | 28,57 | frameshift_variant, feature_elongation |
| 34 | *NRAS* | C>C/T | 1 | 115258747 | snv | 44,93 | missense_variant |
| 3 | *PDGFRB* | C>C/G | 5 | 149514402 | snv | 45,95 | missense_variant |
| 2 | *PHF6* | C>C/T | X | 133527636 | snv | 77,58 | stop_gained |
| 38 | *RAD21* | G>G/A | 8 | 117868927 | snv | 12,23 | stop_gained |
| 28 | *RAD21* | T>T/TA | 8 | 117875443 | insertion | 36,79 | frameshift_variant, feature_elongation |
| 30 | *RUNX1* | G>G/T | 21 | 36252937 | snv | 10,1 | missense_variant |
| 23 | *RUNX1* | C>C/T | 21 | 36252877 | snv | 25,45 | missense_variant |
| 34 | *RUNX1* | C>C/T | 21 | 36231773 | snv | 46,21 | missense_variant, splice_region_variant |
| 19 | *SETBP1* | G>G/A | 18 | 42531913 | snv | 51,01 | missense_variant |
| 12 | *SF3A1* | C>C/T | 22 | 30734818 | snv | 53,83 | missense_variant |
| 23 | *SF3B1* | C>C/G | 2 | 198267359 | snv | 37,6 | missense_variant |
| 38 | *SF3B1* | C>C/G | 2 | 198267359 | snv | 47,09 | missense_variant |
| 29 | *SH2B3* | G>G/A | 12 | 111856661 | snv | 45,42 | missense_variant |
| 29 | *SMC3* | G>G/A | 10 | 112341690 | snv | 31,08 | missense_variant |
| 33 | *SRSF2* | G>G/T | 17 | 74732959 | snv | 34,8 | missense variant |
| 29 | *SRSF2* | G>G/T | 17 | 74732959 | snv | 37,53 | missense variant |
| 38 | *SRSF2* | G>G/T | 17 | 74732959 | snv | 41,85 | missense variant |
| 3 | *SRSF2* | G>G/T | 17 | 74732959 | snv | 43,37 | missense variant |
| 36 | *SRSF2* | G>G/T | 17 | 74732959 | snv | 46,85 | missense variant |
| 34 | *SRSF2* | G>G/T | 17 | 74732959 | snv | 49,69 | missense variant |
| 30 | *SRSF2* | G>G/A | 17 | 74732959 | snv | 49,9 | missense variant |
| 32 | *STAG2* | C>C/CCAAT | X | 123227905 | insertion | 65,85 | frameshift_variant, feature_elongation |
| 34 | *STAG2* | T>T/TATAC | X | 123210268 | insertion | 83,51 | frameshift_variant, feature_elongation |
| 33 | *STAG2* | C>C/T | X | 123220428 | snv | 85,29 | stop_gained |
| 30 | *TET2* | C>C/T | 4 | 106156747 | snv | 38,74 | stop_gained |
| 3 | *TET2* | C>C/T | 4 | 106156747 | snv | 42,66 | stop_gained |
| 38 | *TET2* | GA>GA/G | 4 | 106155754 | deletion | 42,91 | frameshift_variant, feature_truncation |
| 34 | *TET2* | C>C/T | 4 | 106156747 | snv | 44,69 | stop_gained |
| 12 | *TET2* | T>T/C | 4 | 106197285 | snv | 78,53 | missense_variant |
| 2 | *TET2* | C>C/G | 4 | 106193995 | snv | 93,77 | stop_gained |
| 27 | *TP53* | G>G/A | 17 | 7577121 | snv | 16,31 | missense_variant |
| 27 | *TP53* | T>T/A | 17 | 7577084 | snv | 17,18 | missense_variant |
| 25 | *TP53* | C>C/T | 17 | 7577114 | snv | 24,08 | missense_variant |
| 31 | *TP53* | C>C/A | 17 | 7577568 | snv | 31,6 | missense_variant |
| 4 | *TP53* | T>T/C | 17 | 7577536 | snv | 32,26 | missense_variant |
| 17 | *TP53* | C>C/T | 17 | 7577120 | snv | 34,62 | missense_variant |
| 16 | *TP53* | T>T/C | 17 | 7578535 | snv | 34,74 | missense_variant |
| 4 | *TP53* | G>G/A | 17 | 7578212 | snv | 35,33 | stop_gained |
| 16 | *TP53* | C>C/T | 17 | 7577568 | snv | 38,84 | missense_variant |
| 15 | *TP53* | C>C/T | 17 | 7577509 | snv | 39,56 | missense_variant |
| 10 | *TP53* | G>G/A | 17 | 7577022 | snv | 41,03 | stop_gained |
| 10 | *TP53* | C>C/T | 17 | 7577120 | snv | 42,17 | missense_variant |
| 15 | *TP53* | G>G/A | 17 | 7578275 | snv | 42,17 | stop_gained |
| 12 | *TP53* | C>C/T | 17 | 7578413 | snv | 44,99 | missense_variant |
| 14 | *TP53* | C>C/A | 17 | 7577097 | snv | 45,83 | missense_variant |
| 21 | *TP53* | C>C/T | 17 | 7577538 | snv | 48,18 | missense_variant |
| 26 | *TP53* | C>C/T | 17 | 7578406 | snv | 55,46 | missense_variant |
| 9 | *TP53* | T>T/TGGGAA | 17 | 7579388 | insertion | 58,37 | frameshift_variant, feature_elongation |
| 22 | *TP53* | C>C/T | 17 | 7578406 | snv | 67,38 | missense_variant |
| 5 | *TP53* | C>C/T | 17 | 7577120 | snv | 67,47 | missense_variant |
| 11 | *TP53* | G>G/GC | 17 | 7579315 | insertion | 68,59 | frameshift_variant, feature_elongation |
| 6 | *TP53* | C>C/T | 17 | 7577142 | snv | 76,2 | missense_variant |
| 8 | *TP53* | C>C/T | 17 | 7578493 | snv | 90,53 | stop_gained |
| 1 | *TP53* | T>T/C | 17 | 7578190 | snv | 96,15 | missense_variant |
| 18 | *U2AF1* | T>T/G | 21 | 44514777 | snv | 24,07 | missense_variant |
| 17 | *U2AF1* | T>T/G | 21 | 44514777 | snv | 26,71 | missense_variant |
| 32 | *U2AF1* | G>G/A | 21 | 44524456 | snv | 39,67 | missense_variant |
| 35 | *U2AF1* | G>G/A | 21 | 44524456 | snv | 42,42 | missense_variant |
| 14 | *U2AF1* | G>G/A | 21 | 44524456 | snv | 45,5 | missense_variant |
| 12 | *U2AF1* | T>T/G | 21 | 44514777 | snv | 49,79 | missense_variant |
| 28 | *WT1* | G>G/T | 11 | 32456298 | snv | 38,53 | missense_variant |

Chr: chromosome; snv: single nucleotide variant; VAF: variant allele frequency
